# Topology of molecular interaction networks

**DOI:** 10.1186/1752-0509-7-90

**Published:** 2013-09-16

**Authors:** Wynand Winterbach, Piet Van Mieghem, Marcel Reinders, Huijuan Wang, Dick de Ridder

**Affiliations:** 1 Network Architectures and Services, Department of Intelligent Systems, Faculty of Electrical Engineering, Mathematics and Computer Science, Delft University of Technology, P.O. Box 5031, 2600 GA Delft, The Netherlands; 2 Delft Bioinformatics Lab, Department of Intelligent Systems, Faculty of Electrical Engineering, Mathematics and Computer Science, Delft University of Technology, P.O. Box 5031, 2600 GA Delft, The Netherlands; 3 Netherlands Bioinformatics Center, 6500 HB Nijmegen, The Netherlands; 4 Kluyver Centre for Genomics of Industrial Fermentation, 2600 GA Delft, The Netherlands

**Keywords:** Network biology, Review, Molecular biology, Graph theory

## Abstract

Molecular interactions are often represented as network models which have become the common language of many areas of biology. Graphs serve as convenient mathematical representations of network models and have themselves become objects of study. Their topology has been intensively researched over the last decade after evidence was found that they share underlying design principles with many other types of networks.

Initial studies suggested that molecular interaction network topology is related to biological function and evolution. However, further whole-network analyses did not lead to a unified view on what this relation may look like, with conclusions highly dependent on the type of molecular interactions considered and the metrics used to study them. It is unclear whether global network topology drives function, as suggested by some researchers, or whether it is simply a byproduct of evolution or even an artefact of representing complex molecular interaction networks as graphs.

Nevertheless, network biology has progressed significantly over the last years. We review the literature, focusing on two major developments. First, realizing that molecular interaction networks can be naturally decomposed into subsystems (such as modules and pathways), topology is increasingly studied locally rather than globally. Second, there is a move from a descriptive approach to a predictive one: rather than correlating biological network topology to generic properties such as robustness, it is used to predict specific functions or phenotypes.

Taken together, this change in focus from globally descriptive to locally predictive points to new avenues of research. In particular, multi-scale approaches are developments promising to drive the study of molecular interaction networks further.

## Background

Over the last half century, our understanding of life at the molecular level has advanced tremendously. This is made possible by continuously improving technology for measuring the presence or concentrations of molecules at a genome-wide level, such as the microarray (transcriptomics), mass spectrometry (proteomics, metabolomics) and next-generation sequencing (genomics). Perhaps more importantly from a systems biology perspective, similar technology and protocols have been developed to measure interactions among molecules, leading to so-called *interactomics*[1]. Protein-protein interactions are measured using yeast-two-hybrid technology and tandem affinity purification amongst others [2], and stored in a variety of databases [3]; interactions between DNA and proteins, such as histones and transcription factors, are found using yeast-one-hybrid and chromatin immunoprecipitation [4] and deposited in databases such as JASPAR [5] and FactorBook [6]; enzyme-metabolite interactions are measured using enzymatic assays and can be found in for example, BRENDA [7], KEGG [8] and MetaCyc [9]. Besides physical interactions, many indirect interactions have been reported, such as genetic interactions [10], general epistatic interactions [11] and predicted functional interactions [12].

This molecular interaction data is the cornerstone of many computational approaches aiming to analyze, model, interpret and predict biological phenomena, many at a genome-wide scale [13]. Interactions are often thought of as constituting networks, a view already proposed quite early [14] which recently came to full fruition [15]. Networks are now used as vehicles for modeling, storing, reporting, transmitting and interpreting molecular interactions [16]. Often they are represented as graphs, although this is not straightforward for many molecular interactions. For example, metabolic networks, representing physical interactions between enzymes and metabolites as well as conversions between metabolites, are ideally represented by hypergraphs [17] but are often reduced to simple graphs [18] for further analysis.

Although graphs are convenient representations of molecular interaction networks, it was quickly realized that they could be treated similarly to large systems of interacting particles: small sets of interactions might be difficult to understand, but statistical properties relating to all interactions could contain valuable information [19]. This led to **network biology**[20]: a combination of systems biology, graph theory and computational and statistical analyses in which the topology of the graphs representing molecular interaction networks themselves became the subject of study. In subsequent work, statistically maintained properties, such as scale-freeness, were found in molecular networks of different types. In similar analyses, graphs were mined for statistically overrepresented network motifs [21], small subgraphs, suggesting that certain interaction patterns are common to many networks [22].

Despite their apparent universality, it proved difficult to derive biological conclusions from the patterns discovered in these initial global statistical analyses of molecular interaction networks. They may therefore be labeled as *descriptive*, pointing at generic underlying properties rather than leading to verifiable hypotheses. In time, molecular interactions networks were studied more locally, leading to more tangible biological insights. For example, clustering was used to discover significant biological modules and their interconnection patterns, which shed some light on evolutionary constraints of organisms [23]. Ranking of nodes by topological features (such as degree) was shown to relate to biological importance of a gene or protein and may for example be used to prioritize targets for development of pharmaceuticals [24]. We label such approaches *suggestive*. Finally, by studying networks even more locally, typically neighborhoods surrounding a few nodes, it has become possible to derive *predictive* results from molecular interaction networks. A typical approach is to compute a topological fingerprint of the neighborhood around a node; nodes are found to be functionally similar when their fingerprints are similar [25].

Over the past decade, network biology has thus transformed from being an initially descriptive approach to a predictive tool that is routinely applied to discover biologically relevant facts. In this survey, we chart this progression, showing that it corresponds well to a focus change from global to local. Many reviews of developments in network biology have appeared over the last years; here we list those most closely related to ours. Pržulj [26] reviews the use of protein interaction networks in network biology, touching on some of the techniques discussed throughout this review and calling for more integration of biological knowledge with network theory. A review of network theory from the perspective of data mining may be found in Pavlopoulos *et al.*[27]. This review covers a variety of network metrics with an especially strong focus on clustering and node centrality. Likewise, Cho *et al.*[13] review several data-mining approaches applicable to molecular networks. A related topic is that of random molecular networks, which serve as benchmarks against which data mining results are measured. Such networks are generally produced through processes mimicking evolution, several of which are reviewed by Foster *et al.*[28] and Sun & Kim [29]. Finally, many recent reviews focus on the use of network biology in diagnosing disease [30-32], in particular network-based disease markers.

Our review adds to the existing literature by taking a high-level view of network biology as moving from descriptive to predictive, and by maintaining a clear focus on research exploiting the topology of molecular interaction graphs. The remainder of the paper is organized as follows: in Section “Network biology”, a brief overview of relevant biological and mathematical theory is presented. Sections “Descriptive analysis”, “Suggestive analysis” and “Predictive analysis” then give a chronological overview of research on the graph topology of molecular interaction networks, moving from descriptive to suggestive and predictive. We end with a conclusion and outlook in Section “Conclusion”.

## Network biology

For the purposes of this review, we define network biology to be the study of the topology of graph representations of molecular interaction networks, both to describe such networks and as a tool to make biological predictions. We briefly review graph theory and discuss graph representations of molecular interaction networks.

### Graph theory

**Figure 1:**
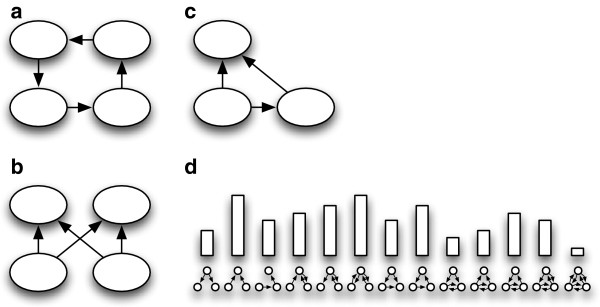
**Some motifs thought to be overrepresented in molecular interaction networks.** Arrowheads indicate link directionality. **(a)** A four-node feed-back motif. **(b)** A four-node bi-fan motif. **(c)** A three-node feed-forward motif. **(d)** Three-node motif signature for a network.

Graph theory is the study of **graphs**: structures representing relationships between pairs of objects [33]. The set  of objects in a graph *G* are called **nodes**; the relationships between the objects are captured by a set  of node pairs called **links**. When nodes *u* and *v* are linked (i.e. {u,v}∈ℒ), *u* is said to be a **neighbor** of *v* and vice-versa. In **directed graphs**, used for modeling non-symmetric relationships such as activation or repression, each link is directed and has a source node (origin) and a target node (destination). The number of neighbors of a node *u* is called its **degree**. Figure 1 shows examples of directed graphs. **Weighted graphs** model non-binary relations by associating scalars or **weights** with links. An example is the affinity with which proteins bind to one another. Table 1 lists some metrics often used to study graphs. Many more metrics in the context of network biology are covered in [27].

**Table 1:** Graph metrics reduce structural properties of network to (vectors of) real numbers, facilitating the comparison of different networks

**Metric types**	**Metric descriptions**
**Degree distribution**	The statistical distribution followed by the degrees of the nodes in a network. Many real-world networks have degree distributions that depart sharply from those of classical random network models (Table 1).
**Path metrics**	In an unweighted graph *G*, the shortest path between nodes *u* and *v* is the minimum number of links one must traverse to move from *u* to *v*. If *G* is weighted, the shortest path is that with the minimal sum of link weights. The **average shortest path** or **characteristic path length** is the average length of all shortest paths (between all node pairs) in a network.
**Centrality metrics**	A centrality metric gives a ranking of nodes according to their “importance”. The simplest measure is **degree centrality** – the degree of a node specifies its importance. **Closeness centrality** is the reciprocal of the sum of the shortest paths to all other nodes (i.e. a node whose closeness centrality is high is close to many nodes). **Betweenness centrality** is the fraction of shortest paths passing through a node. **Eigenvector centrality** and **Pagerank** are measures of how frequently one arrives at a node when performing a random walk on a network.

An **induced subgraph***G*’ of *G* is a subset of the nodes of *G*, along with all links whose endpoint nodes are both in *G*’. In a **bipartite graph**, the nodes can be split into two sets such that no two vertices in the same set are adjacent. A complete bipartite graph in which all nodes from the first set are connected to all nodes in the second is said to be **complete**.

### Molecular interaction networks

**Figure 2:**
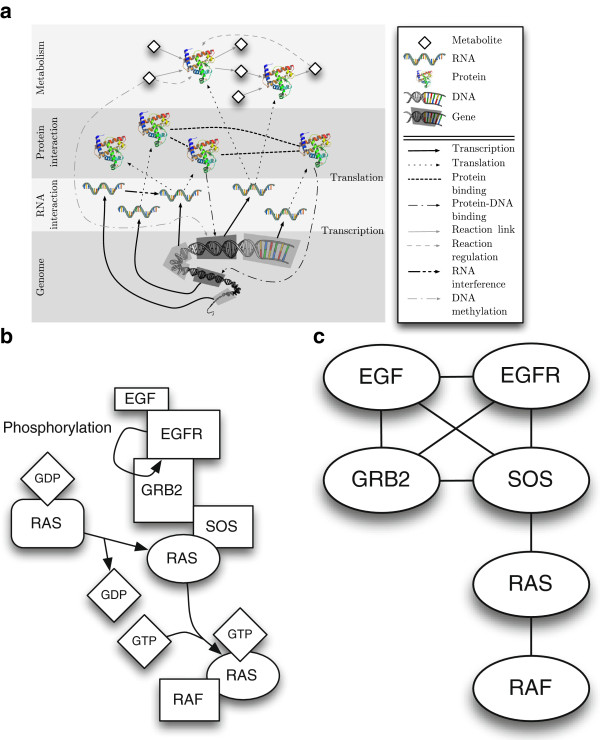
**From biological models to networks.(a)** Simple overview of molecular interactions in the cell. **(b)** Part of the MAPK/ERK pathway modeled as a network. **(c)** Homogenous protein interaction graph representation of part of the MAPK/ERK pathway.

Molecular biology is the study of all cellular processes involving DNA, RNA, proteins and metabolites. A simplified overview of common interactions between these molecules is shown in Figure 2a. Although simplified, models such as Figure 2a are still complex. Researchers generally study models with fewer molecules and interactions, such as the signaling pathway model in Figure 2b.

Both Figures 2a and 2b focus on interactions and can therefore be represented as networks. But neither is a graph, since Figure 2b contains non-pairwise relationships and Figure 2a contains multiple types of relationships while both contain multiple types of nodes. Complex interaction models that distinguish between node and link types are useful when the focus of study is on a small molecular subsystem but a hindrance when the aim is the discovery of *interaction patterns* across large sets of interactions. When pattern discovery is the aim, networks are reduced to graphs by including only links and nodes modeling one or two concepts and by converting non-pairwise links to pairwise links. The graph in Figure 2c is one possible simplification of the pathway in Figure 2b.

While **network** and **graph** are thus two distinct concepts, we will henceforth use the term **network** to refer to both concepts. Table 2 lists several such networks commonly studied.

**Table 2:** Commonly studied molecular interaction networks

**Type of network**	**Network description**
**Association networks**	Association networks model *any* kind of relation between molecules (e.g. binding, co-expression and structural similarities). Examples of association networks are **gene co-expression networks** and **protein similarity networks**.
**Functional networks**	Functional networks model functional relations between pairs of molecules (usually genes or proteins). A link implies that both are involved in the same function, process or phenotype. **Genetic interaction networks** represent interactions where a double mutation leads to an epistatic effect, i.e., worse or better than expected based on the single mutation.
**Protein-protein Interaction Networks (PPI Networks)**	Protein-protein interaction networks are undirected networks that model protein binding. PPI networks are derived from high-throughput experiments using techniques such as yeast two-hybrid screening, mass spectrometry and tandem affinity purification [2]. **Signaling networks** are related to protein interaction networks, but their links are directed according to the flow of molecular signals.
**Transcription-regulatory Networks (TR Networks)**	Transcription-regulatory networks are bipartite networks with one set of nodes representing genes and the other representing transcription factors (TFs). TFs are products of genes (modeled by gene-TF links) whilst genes are regulated by TFs (modeled by TF-gene links). Data for such networks is derived through the process of chromatin immunoprecipitation (ChIP) [34]. **Gene regulatory (GR) networks** are related to TR networks but contain only genes. Their links represent indirect regulatory relationships.
**Metabolic networks**	Metabolic Networks are bipartite networks that model the relationships between the chemical reactions that occur in cells and the substrates involved in the reactions (the solid gray lines in Figure 2a). Reduced, non-bipartite metabolic networks containing only metabolites or only reactions are also often studied.

## Descriptive analysis

During the 1990’s, researchers in various scientific fields started studying macro-scale systems in which individual entities locally interact in simple ways, leading to complex behavior emerging at a global scale. Examples include telecommunications networks [19]**,**[35], social relationship structures [36] and biological interactions from the molecular to the ecological scale [21].

The structure of the above networks departed significantly from the random network models – the Erdős-Renyí-model [37] and the Watts-Strogatz model [36] – commonly used in that day to model large networks (see Table 3). Real-world networks had short average path lengths and degree distributions approximating power laws [19]. The slopes of the degree distributions, when plotted on log- log axes, tended to fall within a narrow range, regardless of the numbers of nodes in these networks. This independence of scale or **scale-freeness** was thought be indicative of networks formed through gradual growth processes based on **preferential attachment**: every time a node is added to a network, it is linked to existing nodes with probabilities proportional to the degrees of those nodes [19]**,**[20].

In biology, initial studies on molecular interaction networks matched the topologies observed in other real-world networks. Gene co-expression networks [44], protein-protein interaction networks [45], metabolic networks [46] and transcription regulation networks [20] all contain aspects of scale-free networks. Nevertheless, although various random network models reproduce some salient properties of molecular networks, each has been criticized for not being consistent with other important aspects of molecular networks [47]**-**[50].

Molecular networks are often also highly clustered, implying **modular** design (see Table 4) and supporting the idea that biological systems are modular at all levels [51]. An early study on the *S. cerevisiae* PPI network showed proteins with similar functional annotations to be highly connected, strongly suggesting modularity [25]. Similarly, in the yeast TR network, highly co-expressed genes were found to be clustered [52]. Evidence for hierarchical modularity was found in a PPI network [53] and in the metabolic networks of several organisms [54]. In general, molecular interaction networks were increasingly thought to consist of modules, linked through connector or linker nodes [55]. In other words, molecular networks are networks of networks that can tolerate disruptions to individual modules but whose functions are sensitive to disruptions module of connectors.

**Table 3:** Classical random network models against which topological characteristics of real-world networks are often compared

**Type of network**	**Network description**
**Erdős-Renyí (ER) **[37]	The oldest class of random networks. To construct a graph instance, links are added between each pair of nodes with probability *p* (a parameter).
**Watts-Strogatz (WS) **[36]	A kind of generalization of ER networks in which links of a regular lattice are rewired. Characterized by high clustering coefficients and short average path lengths.
**Barabási-Albert (BA) **[19]	A class of random networks constructed one node at a time, with new nodes preferentially attaching to existing high-degree nodes. These networks are scale-free (i.e. hub-like) and more closely resemble molecular interaction network networks than ER or WS networks.
**Duplication-divergence**	These networks, inspired by gene duplication and subsequent divergence (in sequence, interaction and function) [38] are generated by duplicating nodes and randomly removing/adding links. Architecturally, duplication-divergence networks are similar to Barabási-Albert networks [39,40]
**Fixed node degrees**	Random networks characterized by their specific node degree sequences that are generated either by randomly rewiring the links of an existing network [41] or through the configuration model [42,43].

**Table 4:** Modules, motifs and graphlets: concepts for decomposing networks into smaller units

**Network decomposition**	**Decomposition description**
**Modules**	are induced subgraphs whose link density is high in comparison to the rest of the graph. This definition is deliberately vague, as what constitutes a module depends on the context and the algorithm used to discover modules.
**Motifs**	are small subgraphs, usually of 3 or 4 nodes, whose over- or underrepresentation may indicate that their structures are important or detrimental to the system [21]. Usually, all distinct motifs in a network are counted, yielding a motif signature for the network that may then be compared to signatures obtained by sampling from an appropriate random network null model (see Table 1) to determine over- or underrepresentation. A signature for all motifs on 3 nodes is shown in Figure 1d. Motif signatures can be used to characterize networks.
**Graphlets**	are similar to motifs but always fully connected. As with motifs, graphlets are used to construct signatures that capture the local characteristics of a network [56].

Although early attempts at understanding molecular interaction networks took a top-down approach, characterizing networks using global metrics such as their degree distributions, it was soon suggested that global behavior of the cell could be the result of local features [57], a bottom-up view. One view was that behavior of molecular interaction networks emerges from the interactions of many small subgraphs or **motifs** (see Table 4), in the same way that the behavior of a computer results from the interactions of simple logic circuits [21]. Statistical overrepresentation of a motif is thought to be evidence that the motif offers a functional advantage to its host organism. Such motifs – feed-back loops, feed-forward loops and bi-fan motifs (see Figure 1) – all have analogues in the electronic world [21]. This fitted well with the increasing popularity of systems biology [58] that advocated an engineering-inspired approach to study biology. Simple motifs may act as sign-sensitive delay mechanisms or as input response-accelerators, depending on their mix of activators and repressors [22]. More complex motifs may even act as logic circuits, switches and memory states, making them interesting building blocks for synthetic biology [59].

Motifs can also be used to characterize networks more globally. Global motif signatures were found to be unique for different types of networks [21] but conserved between organisms [60], providing further evidence that motifs embody underlying design principles in different types of molecular interaction networks, that are preserved across evolution [22].

The global, module and motif views led to the idea that molecular networks are organized at multiple levels of complexity [61]. At the local level, motifs act as small control circuits or building blocks. Motifs aggregate into modules that, through the interactions of their motifs, implement more complex biological processes. At the global level, modules are connected to each other – and may thus exchange information or molecules – through a small number of linker nodes. The fact that certain topological features, such as scale-free degree distributions, are common among molecular networks suggests that the designs of these networks are shaped at all levels by evolutionary mechanisms.

The case for an architecture based on a hierarchy of motifs, modules and global properties was strong and it appeared to be universal, so that its presence came to be assumed. At the local level, overrepresented motifs were used to filter spurious links from noisy high-throughput networks by rejecting links that did not form part of motif structures [62]. At the global level, the assumption of power-law degree distributions led researchers to propose the evolutionary processes of duplication and divergence as leading to preferential attachment in the formation of molecular networks [38].

### Limits to the descriptive approach

Details of the multi-layered view were increasingly disputed as data quality improved and as researchers revisited interpretations of older findings. At the global level, the most contested trait was that of scale-freeness, a property found to arise under many circumstances, challenging its significance [63]. Careful examination of molecular interaction data showed that some non-scale-free distributions fit degree distributions of molecular networks as well as scale-free distributions [64]**,**[65]. More contentious was the suggestion that some global features are modeling artifacts. The hub-like architecture of protein interaction networks was questioned, since no protein can realistically bind to the number of proteins suggested by hub nodes; hub nodes are more likely to represent groups of proteins that only appear to be individuals owing to experimental limitations [47]. Likewise, metabolic networks do not display short average path lengths when metabolite paths are traced; shortest path algorithms on metabolic networks do not take into account the requirement that all metabolites be present for a reaction to occur and their direct application to these networks is meaningless [17].

At the module level, it was found that modules are less clearly delineated than previously assumed. There appeared to be many connections between modules, making it difficult to distinguish linker nodes [66]. Without linker nodes, assignment of nodes to modules is more difficult, leading to “fuzzy” modules. Motifs were also criticized. The bi-fan motif, found to be overrepresented in molecular networks [21] and assumed to be functionally important, was shown to have no characteristic behavior when considered as a dynamic system [67]. If motifs lack characteristic behavior, aggregates of motifs, such as motif clusters, cannot be assumed to implement specialized biological functions. Motif signatures (Table 4 and Figure 1d) of networks were argued to be by-products of simple evolutionary mechanisms (such as gene duplication and divergence) [68]. Evolution may thus not be driven by motifs; rather, motifs may be the inevitable result of the self-organizing effects of evolution.

Although there is less universal structure in molecular networks than once thought, the original multi-layered model is still useful, albeit with some modifications. There is much evidence that molecular networks are not scale-free, but they are generally heavy-tailed [65], meaning that they have a few hubs and many low-degree nodes. Motifs may not be simple biological circuits [21], but they established the idea that local structure is important; one way in which this was later exploited was to compute node signatures for use in function prediction in molecular networks [56] and alignment of molecular networks [69]. Perhaps the most important contribution of the layered view was the idea that molecular networks are organized at multiple levels; the molecular organization of the cell cannot be understood at one scale only.

### Topological features as target or by-product of evolution

The global approach was not meant to be purely descriptive: its original goal was the discovery of universal architectural features. Universality suggests that organisms are selected *because* they posses such features and would provide clues about the topological requirements that are essential to life.

One property thought to emerge from natural selection is *robustness*, the ability to maintain function under perturbations [70]. Network biologists have sought to explain robustness in terms of topological characteristics. In PPI networks, the number of interaction partners of nodes initially appeared to correlate with their essentiality [57]: robustness may come from the fact that PPI networks have few hubs and many low-degree nodes. In metabolic networks, almost the opposite is true, with networks being susceptible to disruption of low-degree linker nodes that connect metabolic modules [71]. However, in both cases the systems are resilient to most perturbations but susceptible to targeted attacks, a property known as *highly optimized tolerance*[72].

After-the-fact attempts to match topology to properties such as robustness were eventually called into question. *In silico* evolution experiments with simple gene-regulatory networks showed that many such structural features emerge from network dynamics rather than selective pressure [73]. Other such network evolution experiments suggested that the drivers could be simple processes such as reuse, genetic drift and mutation [68]**,**[74]**,**[75]. Even higher-level organization such as modularity is thought to arise from such simple processes [23]. A study comparing a metabolic network to a network of atmospheric chemical reactions found large topological similarities and concluded that many large-scale topological features have no functional nor evolutionary significance, the so-called **neutral theory of chemical reaction networks**[76]. In bacteria, horizontal gene transfer is thought to play an important role in module formation, as cells adopt clusters of foreign genetic material wholesale in reaction to environmental variability [77]. Nevertheless, the extent of this influence was recently questioned, stressing possible interplay between variability and gene transfer [78]**,**[79].

Not all network features emerge through network dynamics. Selection pressure does seem necessary for the fine-tuning of topological features and may in some cases be responsible for the difference between a robust and fragile network [80]. In simulations of metabolic network evolution, hubs emerge when networks are selected for their ability to grow [81]. In models of GR network evolution, sparsity (i.e. low link counts) emerges when selectional stability (which models energy minimization of the mutation process) is enforced [82]. Even modularity may rely on selection pressure, albeit in a more subtle form. When networks are evolved and selected for their ability to prosper in varying conditions, modularity is found to emerge and, crucially, to be maintained [83]. A similar result was obtained by subjecting randomly generated metabolic networks (i.e., not generated by a procedure mimicking evolution) to a range of environments and assessing the amount of biomass they produced [84].

## Suggestive analysis

Since the early days of network biology, data mining was used to discover unexpected (ir)regularities in molecular interaction networks. Some findings were already discussed in Section “Descriptive analysis” (the use of clustering to discover functional annotation, the existence of hub proteins). While data mining techniques shed light on aspects of biological function, they do not necessarily lead to directly testable hypotheses. In this sense, we call the methods in this section “suggestive”. We describe four strategies for extracting network regularities: significant feature detection, clustering, central and hub node discovery and network homology.

### Significant feature detection

The idea behind this strategy is that unlikely patterns in molecular networks are indicative of underlying “design” processes (such as evolution). The likelihood of a feature is determined by considering its distribution in network instances generated using a random network model (see Table 1). In early work, PPI networks were rewired (link pairs were shuffled) to generate random networks [41]. The connections between high-degree nodes in the original protein interaction network were found to be statistically unlikely in rewired networks, leading to the hypothesis that interactions between high-degree proteins are suppressed in evolution, perhaps to control cross-talk in the cell.

Modules and motifs [21] can also be considered as significant features. Some of the clustering algorithms mentioned earlier in this section explicitly assess cluster significance as a function of its likelihood [85].

Such significant features can sometimes be biologically interpreted. Statistical analysis of miRNA targets in a human signaling network found that miRNAs tend to target proteins that are part of positive feedback motifs [86]. Similarly, cancer genes tend to be part of positive feedback motifs whilst genes that are highly methylated tend to be part of negative feedback motifs [87]. In both of these cases, the motifs are interpreted as amplification or dampening circuits, analogous to electronic circuits. An interesting recent view is that individual motifs are not necessarily significant but that large clusters of positive or negative feedback motifs act as stochastic amplifiers or dampers, respectively [88].

The advantage of significant feature detection lies in its simplicity: existing techniques are used to analyze and compare the input network and networks derived from a random model. But this is also its main drawback: choosing an incorrect random network model can make features appear significant when they are not.

### Clusters

Modules in complex systems tend to be highly internally connected whilst sharing only a few connections with the outside world. Graph clustering is an approach to discover such modules by decomposing a network into a number of subnetworks or **clusters** that are internally highly connected. The “big data” era has inspired development of clustering algorithms that efficiently deal with large datasets.

In network biology, general clustering algorithms have been used to discover functional modules in gene co-expression networks [89] and genomic cooccurence networks [90]. Since proteins in complexes highly interact with one another, graph clustering has also been used to discover protein complexes in PPI networks [55]. Here we mention a few of such general clustering algorithms; the interested reader is referred to [91] for a more thorough overview. Most modern clustering algorithms are based on physical models, data mining techniques or spatial partitioning. Physics-inspired approaches include spin models [92]**,**[93], random walk models [94]**,**[95] and synchronization models [96]. Data mining approaches treat cluster discovery as a problem of significant feature discovery. A few clustering algorithms discussed below are (at least partially) based on this idea. Spatial partitioning approaches associate distance metrics on pairs of nodes that are then clustered using approaches such as *k*-means clustering. A number of such distance metrics are discussed later in the context of “neighborhood homology” later in this review.

Whilst general algorithms can be applied to molecular networks, clustering algorithms that exploit the specific structure of molecular networks may achieve better results. MCODE is a heuristic algorithm developed to detect complexes in protein interaction networks [97]. Other examples include Restricted Neighborhood Search Clustering [98] and CODENSE, an algorithm for finding dense subgraphs [99]. A number of algorithms based on local neighborhood statistics were proposed as well, for example to find subgraphs of PPI networks that are active according to high-throughput measurements (ActiveModules [100] and MATISSE [101]). More generally, a likelihood score for the density of a subgraph can be used in (greedy) optimization algorithms to mine dense subgraphs, such as in CEZANNE, which finds functional modules in gene co-expression networks [101].

Besides fully connected clusters, clusters that resemble bi-cliques (complete bi-partite subgraphs, see Section “Graph theory”) have been shown to be common and biologically relevant in protein interaction networks [102]. Furthermore, clusters in bipartite networks such as TR and metabolic networks are also manifested as bi-clique-like networks. Algorithms have been proposed to mine such (bi-)clique clusters [103]**,**[104]. Specialized algorithms for bipartite networks have also been developed, such as SAMBA, that integrates additional biological data to discover modules [105].

A still-difficult problem is the discovery of overlapping clusters. Many molecules are components of multiple modules (e.g. proteins are part of multiple protein complexes, metabolites are inputs to multiple metabolic reactions) whilst most existing clustering algorithms place each molecule in exactly one cluster. A relatively simple approach is to group molecules in topics and to apply node-based clustering on the topics; a node that belongs to topics in different clusters would be a member of (at least) two clusters. Recent research uses the more restricted case of edge clustering (which is equivalent to topic clustering on topics of two nodes each) with good success [106]**-**[108].

Clustering is a useful technique to gain understanding of the modular construction of a molecular network, but caution is required. Recovered clusters may not reflect actual biological modules; inaccurate clustering can arise from badly chosen clustering criteria (in particular from criteria unrelated to biological constraints) [109]. Algorithms that produce overlapping clusters may assign nodes to too many or too few clusters and rigorous techniques for handling such problems are still lacking.

### Central nodes and hubs

Early findings in network biology suggested that some nodes are more important or *central*[110] (see Table 2) in molecular interaction networks. This manifestation of highly optimized tolerance entails that the survival of an organism depends more on the presence of a few central nodes than on most other, less central nodes. First, it was found that disrupting the highly connected, “hub-like” p53 gene in the human signaling leads to cancer [111]. It was subsequently shown that the number of interaction partners of a protein (i.e., *degree centrality*) in the *S. cerevisiae* protein interaction network is correlated with its lethality [57]. Research on protein interaction networks [112], co-expression networks [113] and synthetic genetic interaction networks [114] showed similar correlations. Furthermore, the number of interaction partners was shown to be negatively correlated with the rate of evolution in protein interaction networks [115], metabolic networks [116] and transcription-regulatory networks [117], further supporting the idea that central nodes are important.

Closeness centrality was used to find central metabolites in metabolic networks [118]. Betweenness centrality was used to identify bottleneck nodes – nodes of low degree whose removal is fatal to the organism [119]. Both of these metrics fit the interpretation of central nodes as being chemical flow routers. In signaling networks, disruption of central nodes has been linked to cancer, suggesting that they act as information coordinators/routers [120]**,**[121].

However, not all centrality measures can be easily related to routing, examples of which include subgraph centrality [122], coreness centrality [123], bipartivity (the fraction of closed loops including the node that are of even length) [124] and node hierarchy [125].

In spite of the initial positive findings, further experiments on *S. cerevisiae* showed little correlation between protein degree and essentiality [126], a finding strengthened by computer simulations of gene expression [127]. This cast doubt on the use of centrality measures alone to predict node functionality. Some researchers have sought to refine the notion of centrality by considering interaction patterns of central nodes: those that interact with many interaction partners simultaneously are called “party” hubs whilst those that interact with a few of their partners at a time are called “date” hubs [128]. Party hubs are thought to be global coordinators that connect components within network modules whilst date hubs may be local coordinators that connect network modules [128]. However, this distinction has been challenged with the availability of new data that does not show such clear distinctions between central nodes [129].

Even if node centrality is not as well correlated with node function as hoped, research in this field has shown that hubs do tend to be more essential than non-hubs. Furthermore, subversion of central nodes has been implicated in the formation of cancer [120]**,**[130], suggesting possibly useful drug targets.

It has been suggested that a simple explanation for the essentiality of high degree nodes is that they are more likely to interact with essential complexes and their removal breaks such complexes [126]. The implication is that local topology is a deciding factor in essentiality. Indeed, versions of existing centrality measures modified to take more local information into account are better at predicting which nodes are essential [131]. However, it is important not to conflate node essentiality, a concept tied to survivability, with the influence that a node exerts on a network. The latter concept is discussed in the next section in the guise of “controllability”.

### Global homology

The principle of homology states that biological systems related by evolution are structurally similar. Its converse – structural similarities imply common heritage – is often used to predict the function of unknown proteins and genes. In networks, topological similarity can likewise be used to infer functional similarity. Using this approach, metabolic networks of 43 organisms were found to display hierarchical modularity [54]; these modules were found to center around core metabolites [132]. In the same vein, the connectivity of a protein in a PPI network was shown to be proportional to its age. In a study on three species, common proteins are likely to be older than those present in only a single species [133].

The approaches above focus on high-level similarities between networks without attempting to match individual nodes in the networks. By performing such alignments, clustering and significant feature detection applied across species can lead to more insight. In an early example, the glycolytic pathways of 17 organisms were aligned [134] and revealed many interesting differences between species in this essential part of metabolism. Alignment of the *E. coli* metabolic network to those of other organisms identified enzymes whose genes were candidates for horizontal gene transfer [40]. The average degree of these candidates is higher than that of other enzymes, implying that they are central to metabolism. Thus, ancestors to *E. coli* replaced their central enzymes with better functioning enzymes from other species.

### Data mining in biological networks suggests biological findings

Data mining techniques have been successfully applied in network biology to suggest biological functions for genes and proteins. The common theme is that instead of considering global properties of biological networks, they focus on subnetworks, from individual nodes to neighborhoods and features shared between networks. This increased focus allows the derivation of more tangible biological results. However, when analyses are based on comparisons to random network models (Table 1), such as in significant feature detection, the problem of telling these apart from evolutionary by-products remains.

## Predictive analysis

The data mining approaches discussed in Section “Suggestive analysis” reveal the large-scale organization of molecular networks in some detail but do not, in general, yield testable biological hypotheses. Approaches that do give such results tend to be based on network generalizations of existing principles in molecular biology: guilt-by-association, homology and differential analysis.

### Guilt-by-association

The principle of guilt-by-association is based on the observation that if most of the interaction partners of a molecule are associated with some property (such as a specific biological process or molecular function [135]), the molecule itself is also likely to be associated with that property [136]. Guilt-by-association has been used to assign functions to proteins with unknown roles based on the functions shared by the majority of their direct neighbors (i.e. interaction partners) in protein interaction networks [25]. The properties shared by the majority of a node’s neighbors do not necessarily yield the best annotations [137] and more sophisticated approaches, such as Markov random fields trained on node neighborhoods [138], have been developed as alternatives.

By only taking direct interactions into account, the above applications of guilt-by-association ignore the impact of potentially informative indirect interactions. So-called *n*-hop features have been used to predict disease associations of proteins in PPI networks [139]. Another technique for incorporating indirect neighbors is graph diffusion, an idea derived from the study of diffusion in physical systems. Here, properties of nodes are diffused across links in a network; properties that diffuse in high quantities to nodes with unknown roles are used to annotate these nodes [140]. In both *n*-hop methods and graph diffusion, interaction strength between nodes depends on the path structure between the nodes.

Path structure need not be the only determinant of interaction strength. Nodes that are members of the same biological module may have similar functions [25]. Thus, a node whose role is unknown can be annotated with the functions appearing most frequently in the module(s) to which it belongs. Whilst we do not know what the biological modules are, we can compute approximate modules through clustering. Such an approach has been used to annotate unknown proteins in *S. cerevisiae* protein interaction networks [103]. Guilt-by-association is a simple and effective technique that extends naturally to networks. However, it is only effective when the roles of the majority of molecules in a network are known, limiting the technique to well-studied organisms.

### Neighborhood homology

Since the use of homology is pervasive in biology, we expect the principle to extend to networks. Indeed, in Section “Global homology” it was already discussed how networks found in different organisms have similar structural properties. Predictive approaches use topological and possibly biological similarity to match similar nodes across different networks. Once nodes are aligned, the function of a protein or gene whose role is unknown can be predicted, if the function of its matched node in the other network is known.

The first network alignment algorithms operated at a local level, attempting to match only small parts of entire networks to one another [69]**,**[141]. Global alignment is more difficult, because networks to be aligned generally differ in size. Moreover, homology is not a one-to-one relation: many nodes may align to many nodes. There are two main approaches for performing global alignment: 

1. Cluster the nodes in each network and compute topological matching scores on the clusters [142]**,**[143] (“matching clusters”).

2. Select groups of nodes in different networks that are pairwise similar in local neighborhoods and possibly biological labels [144]**,**[145] (“clustering matches”).

The first type of algorithm has the disadvantage that the clustering step precedes matching and thus ignores potentially useful information. Many algorithms of the second type associate feature vectors of topological (and possibly biological) attributes with nodes that are then used to compute node similarity. Various metrics have been used [146]. The Jaccard coefficient, a measure of overlap between sets of binary attributes, has been widely used, an example of which was the prediction of protein function in human PPI networks [147]. The *h*-confidence metric [148] is a data-mining tool for discovering associations and has been used in protein function prediction. Specialized metrics, such as the graphlet distance (tailored to graphlet signatures[56]) have been used to discover genes implicated in cancer [149].

Variations of clustering algorithms, looking for dense subgraphs within one network, have been proposed to mine subgraphs similar in two networks. For example, the PathBlast algorithm combined a statistical score for protein similarity and probability of a reported protein interaction to mine pathways or complexes occurring in PPI networks of different species [141]. Similar approaches were applied to assign functions to proteins [150] and to align metabolic pathways [151].

### Differential analysis

Diagnosis of many diseases (such as cancer) is based on the fact they influence the regulation programs of cells. Traditionally, this involved finding changed expression of marker genes, or specific gene mutations, i.e. focusing on the nodes in the network. Network biology allows additional focus on node relations, making it possible to diagnose molecular diseases that cannot be well characterized by the traditional techniques [152]. This so-called differential analysis, finding changes in network structure [31], is currently complicated by the fact that construction of high-quality molecular networks requires considerable time and resources. One common way around this is to use an existing high-quality network, typically a PPI or TR network, as a scaffold onto which noisy high-throughput patient data (typically gene expression or methylation data) is overlaid. If multiple measurements are available for each patient, gene coexpression/comethylation values can be computed and overlaid as link weights on PPI links.

Expression changes of genes/proteins linked to central nodes in molecular networks have been proven to be reliable markers of disease. Differential expression around topologically central nodes in protein interaction networks has been used to diagnose cancer [153]**,**[154]. Disease central nodes (i.e., nodes implicated in disease) have been similarly used in the diagnosis of breast cancer and leukemia [155]. More recently, co-expression changes around biologically central nodes, such as signaling hubs, have shown to be even more reliable disease markers [156]**,**[157].

More elaborate differential approaches consider changes in expression patterns of subnetworks, instead of only central nodes. Automatic extraction of such subnetworks based on topology and measurements such as gene expression has revealed subnetworks associated with cancer (in which differential gene/protein expression could be used for diagnosis of the disease) [87]**,**[158] as well as subnetworks that are implicated in heart failure [159]. An alternative to automatic extraction is to use biological modules based on theoretical knowledge; such an approach has been used in cancer prognosis [160].

Differential diagnosis, despite its relative newness has quickly grown to a large field. Our discussion is necessarily limited by the scope of this review; the interested reader is referred to recent reviews that consider the discipline in much more depth [31]**,**[32]**,**[161].

### Relating topology to biological properties leads to predictive power

The data mining techniques discussed in Section “Suggestive analysis” are mostly based on topological information. In contrast, the predictive approaches discussed above depend on additional biological information. This approach to network biology clearly yields more testable hypotheses than the suggestive and descriptive approaches.

Since we do not, in general, have good models of biological function at large scales, predictive approaches are most often applied to small groups of nodes or subnetworks. There are exceptions with metabolic networks being the most prominent. Flux balance analysis (FBA) [162]**,**[163] is a framework for computing steady-state reaction rates in such networks based on reaction stoichiometry, assuming the cell attempts to achieve some objective such as maximum growth. FBA is often used in a predictive way, but has also been applied in a “suggestive” setting, e.g. to study robustness of metabolic networks [71]. FBA allows one to take additional physical constraints into account, such as thermodynamic interactions [164] or responses to signaling [165]; for an extensive overview see [166].

The biggest problem with incorporating additional biological knowledge into existing models is that, for any given biological attribute, we seldom have complete data. Two recent ideas, “controllability” and “observability”, potentially allow to use partial (local) knowledge to predict global state. Controllability refers to “driver” nodes that have a large influence on the state of a system [167]; observability is almost complementary, focusing on a small set of appropriately chosen observation nodes whose properties allow reconstruction of the global system state [168]. These techniques promise to allow associating local information with driver/observation nodes and to predict global properties from limited available data.

## Conclusion

In this review, we have summarized common research themes in the field of network biology. We find a slow movement from global to local analysis, arguing that this trend emerged from a need to draw more concrete biological knowledge from networks.

The survey findings seem to suggest that one must either choose between untestable abstract hypotheses about large-scale topological patterns or small-scale results that neglect large-scale topology. But the successes of local techniques lie not in their focus on the local but because *they tightly couple topological observations to biological knowledge*. From this starting point, we see two broad research directions for improving the explanatory power of large-scale topology patterns. The first approach is theoretical and is aimed at making descriptive and suggestive techniques more predictive, whilst the second approach is practical and extends the predictive techniques to work at larger topological scales.

The theoretical research direction entails the improvement of network evolution models in order that they reproduce as much of the topological aspects of real molecular networks as possible. Better models of network evolution can better reveal the topological features that are by-products of evolution, permitting researchers to concentrate on explaining topological results that cannot be explained by the models. An additional benefit is that these models could themselves lead to biological insight.

In the practical direction, we propose the application of predictive techniques to various “resolutions” of molecular networks, that is, multi-resolution analysis. Lower resolution versions of a network are typically obtained by grouping subnetworks into meta-nodes (by analogy, the entire street network of a city is represented by a single city node in national road maps). How nodes are grouped depends on the topological properties that must be maintained in low-resolution network versions. Node clustering techniques from Section “Suggestive analysis” can be used to produce low-resolution networks by grouping node clusters into meta-nodes. Another promising technique that aims to maintain random-walk properties is **spectral coarse graining**[169].

The two research directions outlined above are by no means the only possible paths for developing network biology. Rather, they show this young field still has much potential for development; we expect that future researchers will bring us unexpected biological insights with the help of network biology.

## Competing interests

The authors declare that they have no competing interests.

## Authors’ contributions

WW performed literature research and drafted the manuscript together with DR. PVM, HW and MR helped draft the manuscript. All authors read and approved the final manuscript.
